# Nanomaterials-modified reverse osmosis membranes: a comprehensive review

**DOI:** 10.1039/d4ra01796j

**Published:** 2024-06-12

**Authors:** Mahmoud A. Ahmed, Safwat A. Mahmoud, Ashraf A. Mohamed

**Affiliations:** a Chemistry Department, Faculty of Science, Ain Shams University Cairo-11566 Egypt mahmoudmahmoud_p@sci.asu.edu.eg; b Veolia Water Technologies Cairo 11835 Egypt; c Physics Department, Faculty of Science, Northern Border University Arar 13211 Saudi Arabia

## Abstract

Because of its great efficiency and widespread application, reverse osmosis (RO) is a popular tool for water desalination and purification. However, traditional RO membranes have a short lifespan due to membrane fouling, deterioration, decreased salt rejection rate, and the low water flux with aging. As a result, membrane modification has received a lot of attention recently, with nanomaterials being extensively researched to improve membrane efficacy and lifespan. Herein, we present an in-depth analysis of recent advances of RO membranes modification utilizing nanomaterials. An overview of the various nanomaterials used for membrane modification, including metal oxides, zeolites, and carbon nanomaterials, is provided. The synthesis techniques and methods of integrating these nanomaterials into RO membranes are also discussed. The impacts of nanomaterial change on the performance of RO membranes are addressed. The underlying mechanisms responsible for RO membrane enhancements by nanomaterials, such as improved surface hydrophilicity, reduced membrane fouling *via* surface repulsion and anti-adhesion properties, and enhanced structural stability, are discussed. Furthermore, the review provides a critical analysis of the challenges and limitations associated with the use of nanomaterials to modify RO membranes. Overall, this review provides valuable insights into the modification of RO membranes with nanomaterials, providing a full grasp of the benefits, challenges, and future prospects of this challenging topic.

## Abstract 2

Because of its great efficiency and widespread application, reverse osmosis (RO) is a popular tool for water desalination and purification.

## Graphical abstract

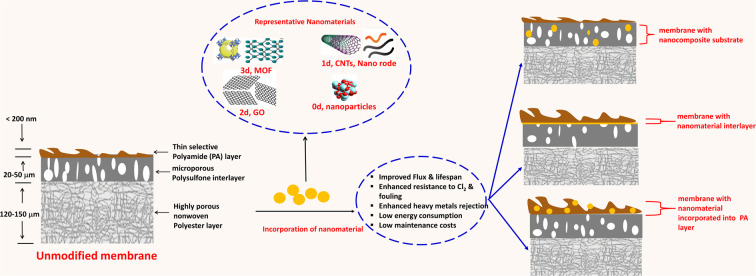

## 1. Introduction

The global water crisis threatens human beings, economies, and ecosystems worldwide. Access to clean and safe water is a fundamental human need, yet a significant portion of the global population still lacks this vital resource. Population expansion, urbanization, climate change, water pollution, unsustainable agricultural practices, inadequate water infrastructure, and water conflicts all contribute to the global water crisis. Understanding the complexities of this crisis and developing innovative mitigation strategies is crucial for ensuring water security and sustainable development. Scientists and researchers are constantly looking for sustainable and cost-effective tools to obtain clean and potable water.^1,2^ The majority of the Earth's water, approximately 97%, is found in the form of saline water in oceans and seas, making it a vast and untapped resource that could help alleviate the water crisis.^3^ Consequently, desalination has emerged as a highly efficient and cost-effective solution to the global water scarcity problem. However, in areas where access to brackish or seawater is limited, wastewater reclamation provides a viable solution to the water crisis.^4^ RO desalination has become a widely adopted process for producing clean water, with numerous sources contributing to its feed, with about 60% coming from seawater, 20% from brackish water, and the remaining from other sources including rivers, and wastewaters.^5^

Desalination methods can be categorized into two main groups based on their separation mechanisms: phase-change and membrane-based processes. Phase-change methods involve the continuous cycling of evaporation and condensation to eliminate salts from water and are characterized by high capital and operational costs and are acknowledged for their intensive energy consumption, mainly reliant on thermal energy derived basically from fossil fuels.^6–9^ On the other hand, membrane-based desalination relies on specialized membranes, which act as filters to allow water to pass through while retaining salts and minerals, *e.g.*, reverse osmosis, forward osmosis, nanofiltration, electrodialysis, membrane distillation, and capacitive deionization methods.

**Fig. 1:**
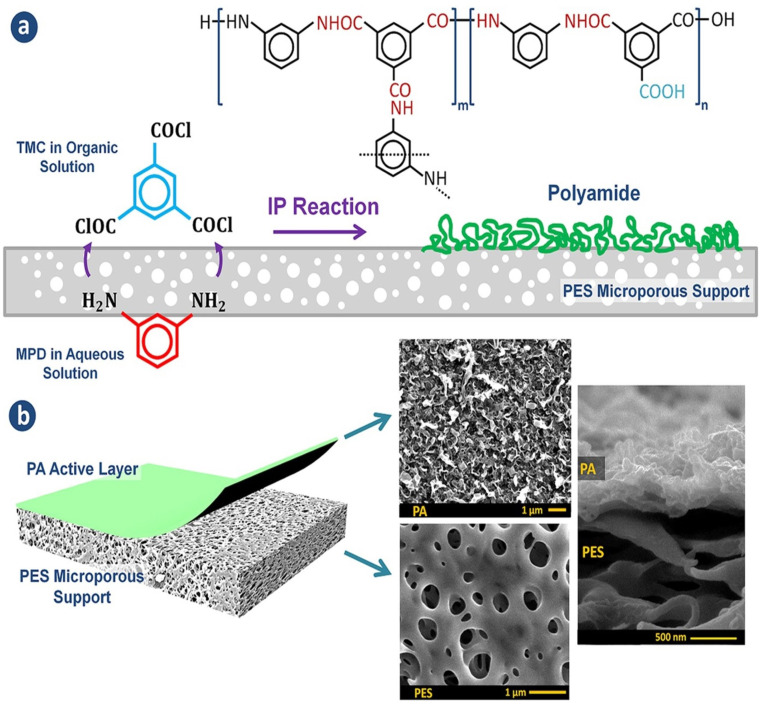
(a) The interfacial polymerization process between TMC and MPD on a microporous support layer. (b) Structure of the fabricated TFC membranes with the top and cross-sectional morphologies.^148^

Currently, RO processes are the most widely used, accounting for over 65% of desalination plants globally. This is attributed to its simplicity, low-energy consumption, separation efficiency, stable product quality, and relatively low operational costs. The first thin-film composite (TFC) membrane was develop using an interfacial polymerization reaction,^10^ such as the combination of trimesoyl chloride (TMC) and *m*-phenylene diamine (MPD) or piperazine (PIP), as shown in Fig. 1a.^11^ A conventional polyamide (PA)-based RO membrane typically comprises three layers: a very thin active layer made of aromatic PA, a support layer with small pores, and a layer of nonwoven fabric, as shown in Fig. 1b. These layers have approximate thicknesses of 200 nm, 20–50 μm, and 120–150 μm, respectively.^12,13^ The polyester support layer alone cannot offer a suitable surface for the PA active layer due to its uneven and porous structure. To overcome this issue, a microporous polysulfone interlayer is put between the selective layer and the support layer.^14^ This extra layer protects the ultra-thin selective layer from high-pressure compression.^15^ The PA layer is responsible for providing selectivity, with typical salt rejection of about 99%.^16^ TFC membranes have a high selectivity and water flux; however, its use in RO processes faces several challenges that significantly impact their overall performance.^17^

**Fig. 2:**
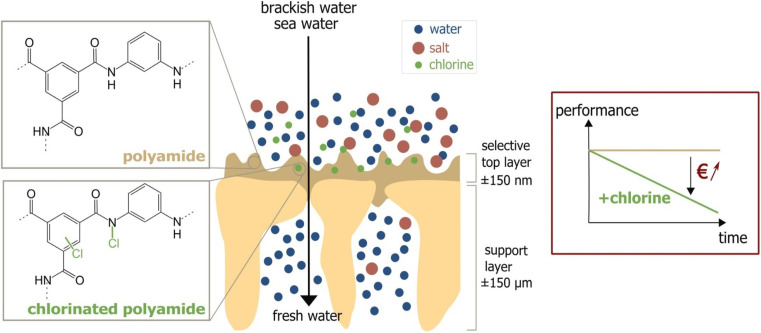
Chlorine attack on PA membrane and the performance decline due to the attack. Reprinted with the permission of ref. 23, copyright 2024, 2024 Elsevier.

One major compromise that RO membranes face is the trade-off between membrane permeability and salt rejection/selectivity. Finding a balance between high permeability and excellent salt rejection/selectivity is crucial in designing efficient RO membranes. Furthermore, fouling is a persistent issue that degrades RO membranes' performance. Membrane fouling occurs when organic substances, mineral scales, colloids, and biofilms accumulate on the membrane's surface or within its pores.^18^ This process is complex and is controlled by factors such as the membrane's surface hydrophilicity, roughness, and charge, as well as feed water quality and operational parameters.^19,20^ A more hydrophilic and smoother membrane is less susceptible to fouling as it discourages foulants from attaching.^21^ Hydrophilic materials are more resistant to fouling because they can undergo hydration, mainly *via* hydrogen bonding. Conversely, surface roughness provides more area and surface defects for foulants to adhere to membrane surfaces, increasing the likelihood of fouling.^22^ Managing these variables is critical for minimizing membrane fouling. One of the most convenient approaches for preventing biofouling in RO units is the chlorination of feed water.^23^ However, the use of chlorine can have detrimental effects on the polyamide barrier layer, which is a critical component of the TFC-RO membrane.^23^ Chlorine attacks the polyamide layer through several mechanisms Fig. 2.^24^ One mechanism involves the substitution of the hydrogen in the amide group with a chlorine atom.^25^ Further, chlorine can cause rapid *N*-chlorination followed by intramolecular rearrangement, leading to the migration of chlorine to the aromatic ring.^26^ Another mechanism is the direct replacement of chlorine atoms for the aromatic ring of *m*-phenylene diamine.^24,27^ Moreover, chlorine promotes the hydrolysis of the amide group, resulting in the formation of amido and carboxylic groups. Oxidation of the membrane by chlorine degrades performance, specifically water permeability and salt rejection.^28,29^

Therefore, to address the challenges that RO membranes face, scientists have extensively researched the use of numerous materials and strategies to enhance membrane properties. Among these, the incorporation of nanomaterials derived from metal oxides, zeolites, and carbon-based nanoparticles into RO membranes have emerged as intriguing enhancement option. Nanomaterials have been proved to improve multiple aspects of RO membranes, including water permeability, chlorine resistance, antifouling potential, and antimicrobial characteristics.^30^ Furthermore, nanomaterials have been found to improve the mechanical strength and thermal stability of RO membranes. This performance enhancement can be attributed to mechanisms such as increased surface area, improved interfacial interactions, alteration of surface features, and enhanced structural integrity provided by the incorporated nanoparticles.^31–33^ For instance, carbon-based nanomaterials, including graphene, carbon nanotubes, and metal organic frameworks (MOF) have showed potential in improving RO membrane performance. These nanomaterials possess high surface area, and unique porosity and adsorption capabilities. They can act as molecular sieves, allowing water transport while effectively excluding salts and pollutants. Carbon-based nanomaterials, for instance, can reduce fouling potential and extend membrane lifespan. Additionally, carbon-based nanomaterials have excellent mechanical strength and chemical stability, which improve the durability and lifespan of the modified membranes. They can withstand harsh operating conditions, such as high pressures and varying pH levels, making them suitable for industrial-scale RO applications. Further, the microporous structure of zeolite nanoparticles selectively allows water molecules to flow through while hindering the transport of larger molecules, assuring effective rejection of pollutants and reducing the likelihood of fouling.^34^ Furthermore, metal oxide nanoparticles can exhibit photocatalytic properties, enabling the degradation of organic pollutants that contribute to fouling. This photocatalytic activity effectively reduces fouling and maintains the membrane's permeability and salt rejection capabilities over prolonged operation. Moreover, Silica's nanoparticles remarkable affinity for water makes it an appealing material for improving the hydrophilicity, water permeability, and efficiency of RO membranes.^31^

**Fig. 3:**
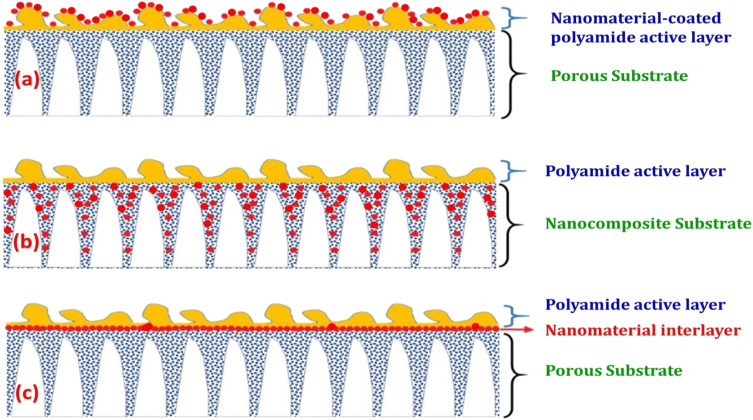
Schematic illustrations demonstrating typical structures of nanomaterial-modified PA TFC membranes: (a) TFC membrane with nanomaterial-coated PA surface layer, (b) TFC membrane with nanocomposite substrate and (c) TFC membrane with nanomaterial interlayer. Reprinted with the permission of ref. 32, copyright 2024, 2024 Elsevier.

**Fig. 4:**
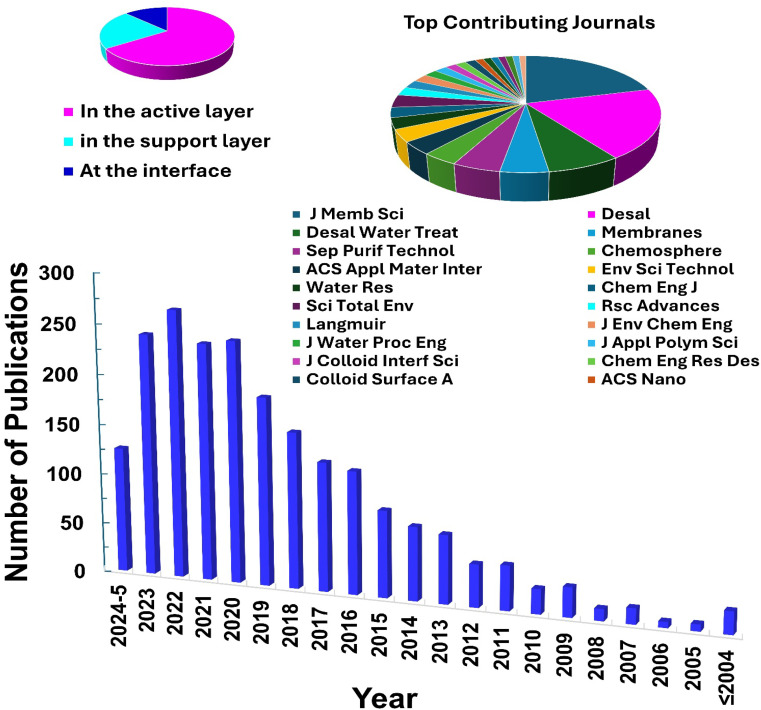
Number of publications related to nanomaterial-modified FN RO membranes (retrieved from Scopus keywords “(reverse osmosis OR RO) AND (nanoparticles OR nanomaterial OR nanocomposite)” on 27th May 2024). Insets show top contributing journals to the topic and the position of the nanofiller in the thin film nanocomposite.

The characteristics of nanocomposite membranes undergo significant alterations based on the nature, concentration, chemical properties, and size of the incorporated nanomaterials.^33^ Consequently, it is possible to customize the properties of nanocomposite membranes according to the specific nanomaterial employed.^33^ Nanomaterials can be integrated into thin film composite (TFC) membranes through various primary approaches, as shown in Fig. 3.^33^ Firstly, nanomaterials can be dispersed within the organic/water phase of polyamide (PA) monomers during their interfacial polymerization (IP) reaction whereby the nanomaterials become randomly captured and enveloped by the active PA matrix, resulting in TFNa membranes (the suffix “a” means: within the active PA layer).^35,36^ Alternatively, nanomaterials can be dispersed within the substrate matrix through the process of phase inversion, resulting in the formation of TFNs membranes with nanocomposite substrates (the suffix “s” means: within the substrate).^37^ Lastly, nanomaterials can be uniformly coated or deposited onto the substrate surface before the IP reaction, yielding TFNi membranes with nanomaterial interlayer (the suffix “i” means: at the interface of substrate).^38^ These various incorporation methods play a significant role in the development of TFC membranes and are important aspects to consider when examining their performance. The escalating interest in the advancement of nanomaterial-modified RO membranes is clearly apparent from the growing number of research studies published, as demonstrated in Fig. 4. This Figure shows the major contribution of TFNa over both TFNs and TFNi membranes, as well as the major contributing journals to this interesting topic. This observation underscores the necessity to analyze and review the existing literature on this topic.

This review offers a thorough and in-depth analysis of recent advancements in the field of nanomaterials-modified RO membranes. The article systematically explores the synthesis methods employed to incorporate carbonaceous and inorganic-based nanomaterials into membranes, along with the characterization techniques used to assess their properties. Moreover, in-depth characterization methods for evaluating the structural, morphological, and physicochemical properties of modified membranes will also be addressed. The impact of these modifications on critical performance parameters, such as water permeability, salt rejection, fouling resistance, and membrane stability, will be discussed in detail. This review will also address the challenges associated with scaling up these modifications from the laboratory to the industrial scale and will identify future research directions to promote further advancements in the field. By critically analyzing the existing literature and providing valuable insights, this review aims to contribute to the development of innovative and effective RO membrane technologies for sustainable water treatment.

## 2. Nanomaterials and nanocomposites-enhanced polymeric membranes

Various nanomaterials can be used as fillers to enhance water permeability and salt rejection rates of polymeric TFC membrane for NF and RO applications. These nanofillers can be tailored based on various factors that operate collectively, including composition, size, shape, porosity, hydrophilicity, the interfacial polymerization method employed, and the membrane layer where these nanofillers have been incorporated, as well as its preparation method. Various nanofiller shapes have been identified including nanospheres, nanotubes, nanosheets, nanocrystals, nano-bowl, nano-cubes, hollow particles, and core shell shaped nanoparticles.^39,40^ Moreover, the researchers interestingly compared the performance of non-porous and porous nanofillers having various compositions, sizes, inclusion membrane layer, interfacial polymerization process, and operated under different conditions.^39,40^

Furthermore, the effect of nanofiller positions on TFC membrane performance was interestingly established.^41^ Membranes can be classed as TFNa, TFNs, or TFNi based on where nanomaterials are incorporated, *i.e.*, in the active layer, the support layer, or at the interface between the two. The researchers found that TFNa membranes for RO and FO applications and TFNi membranes for NF were more effective in overcoming the trade-off between water permeability and selectivity. Furthermore, compared to conventional nanomaterials, the use of porous 1D or 2D nanofillers, such as nanosheets, nanotubes, hollow and mesoporous NPs, in the fabrication of TFNi and TFNs membranes gave superior water permeability through their pores and channels, as well as enhanced selectivity due to their hydrophilicity.^41^ Nevertheless, nano-bowl-shaped nanofillers with concave cavities reduced mass transfer resistance of water molecules and shortened their flow-through pathways. It is worth noting that these factors were not studied using monovariate optimizations due to the practical limitations involved in researching each factor separately while keeping all other factors constant. The current review discusses some representative examples of specially tailored nanofillers with fruitful characteristics for their potential performance in modifying TFC membranes.

### 2.1 Carbon nanomaterial

Carbon based nanomaterials are attractive for membrane modification owing to their porous structures, biocidal activities and hydrophilic properties. A variety of carbon nanomaterials, such as carbon nanotubes (CNTs), graphene oxides (GO) and metal organic framework (MOF) have been used to modify various membranes. Table 1 Shows the performance evaluation of several TFN reverse osmosis membranes with incorporation various carbon-based nanoparticle.

**Table 1:** Performance evaluation of several TFN reverse osmosis membranes incorporating various carbon-based nanomaterials

Modifier type	Test conditions TDS (ppm), temperature, pressure	Flux L m^−2^ h^−1^	Rejection %	Remarks	Ref.
GO	2000, 25.1, 25 °C 20.6	39 to 59.4	95.7 to 93.8	GO nanosheet interlayer spacing has acted as water conduits, increasing water permeability in the process	149
CNT	2000, 25 °C, 15.5 bar	40.9 to 51.3	>97	The mechanical properties of the polymer nanocomposite are enhanced by the incorporation of CNTs	90
				Due to its smoother surface, TFN RO membrane exhibits less fouling potential and improved cleaning recovery efficiency	
Oxidized MWCNTs	2000, 25 °C, 15.5 bar	20.3 to 28.9	>97.4	By including oxidized MWCNTs in the polyamide layer of the RO membranes, the fouling resistance was increased	150
CNT	2000, 25 °C, 15.5 bar	37.2 to 43.5	97.5 to 95.4	The hydrophobic nanochannels of CNTs and good-dispersed states in the PA formed through interactions between PA and CNTs are credited with the excellent membrane performance (high salt rejection and water flux) and enhanced stability of the PA membranes containing CNTs	89
MWNTs	2000, 25 °C, 15.5 bar	14.8 to 28.05		The results demonstrated the ability he electron-rich MWNTs for protection of active sites in MPD form attack by chlorine	56
GO	1000, 25 °C, 15.5 bar	45 to 59.5	>97	For the duration of the assessed exposure time, GO layer increases chlorine resistance	40
GO	2000, NA, 20 bar	17.2 to 31.8	97.9 to 98.7	GO improve resistance of the membrane towered chlorine by preserving its structure. Moreover, the results showed that the enhancing antibacterial activities of RO PI-GO TFN membranes due to oxidative stress mechanism	151
UiO-66	2000, 25 °C, 15 bar	36.7 to 56.8	99 to 99.35	The incorporation of UiO-66 into the membrane showed a marginal increase in rejection and ∼a 50% increase in water flux compared to the pristine membrane under the brackish water condition. Further, in a simulated seawater test, the UiO-66 modified membrane enhanced the boron rejection by ∼11% owing to its significant porous structure which gives extra pathways for water movement, as well as powerful adsorption to boron which leads to further pore narrowing impact	152
ZIF-8	2000, 25 °C, 15.5 bar	40.8 to 61.2	98.9 to 99.2	The chemical alterations and the contribution of extra water transport channels made possible by ZIF-8 inclusion, according to the obtained results, are responsible for the increase in water permeance	102
ZIF-8	2000, NA, 15.5	19.8 to 52.3	98.1 to 98.5	Compared to the pure PA surface, the TFN surface was less crosslinked and more hydrophilic. For the impacts of filler on the shape and characteristics of the TFN membrane surface, an encapsulating mechanism was proposed	153
ZIF-8	2000, NA, 15.5 bar	13.9 to 21.3	98.4 to 99.4	The results demonstrated that ZIF-8 can significantly improve the polyamide fouling resistance and, in this study, the modified membrane reduced the fouling by more than 75%	154
MIL-101 (Cr)	2000, NA, 16 bar	24.3 to 35.2	99.3 to 99.1	MIL-101 (Cr)'s porous structures can provide direct water channels for water molecules to go through fast in the thick selective PA layer, enhancing the membranes' water permeance	101

#### 2.1.1 Graphene and graphene oxide

**Fig. 5:**
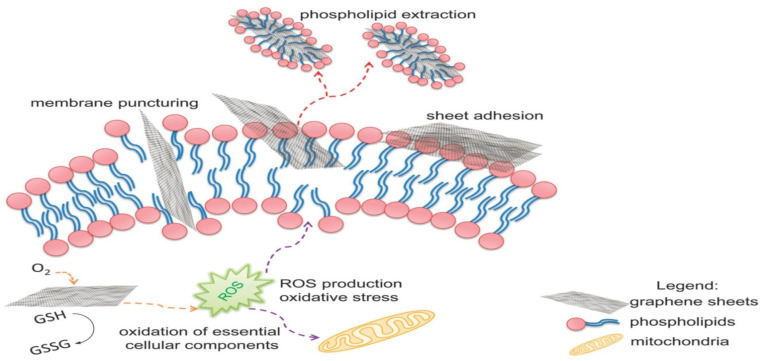
The proposed antimicrobial mechanisms of GO. Reprinted with the permission of ref. 55, copyright 2024, RSC.

Graphene (G), a thin sheet and smooth two-dimensional nanomaterial composed of carbon atoms arranged in a hexagonal lattice, has garnered significant attention in the field of materials science. Its remarkable thermal, mechanical, and electrical properties have positioned it as a highly promising material.^2,42–47^ This has led to the emergence of graphene-based nanomaterials, including graphene, reduced graphene oxide (rGO), and graphene oxide (GO) which have found widespread applications in various applications, such as electrochemical processes, photocatalysis, and sensors.^43,48,49^ Water desalination has been a particular focus for graphenes in membrane separations due to its atomically smooth and thin structure. Graphene's hydrophobic characteristics and atomic-level smoothness allow water to flow with minimal friction between the graphenic layers. This unique feature enhances water transport through the membrane, making it desirable for desalination processes.^50–52^ However, GO and rGO nanomaterials are more hydrophilic than graphene itself and exhibit antifouling and antimicrobial properties that are advantageous for thin-film composite membranes used in desalination. Long-term contact between microbes and GO or graphene sheets leads to disruptions in cell membrane integrity and oxidation of cellular components, ultimately leading to a loss of cell viability.^53,54^ Additionally, the hydrophilic nature of GO and rGO nanoparticles contributes to anti-adhesive features on the membrane surface, which reduces foulant deposition. Oxidative stress, penetration *via* lipid bilayers, and lipid extraction *via* graphene sheets are further potential interactions (Fig. 5).^55^

Functional groups, such as carboxyl, hydroxyl, and epoxide groups, present on GO nanosheets enhance the negative charge of the membrane surface. This strengthens the electrostatic repulsion between extracellular polymeric substances (EPS) and microorganisms, decreasing microbial adhesion to the membrane surface.^56^ The presence of hydrophilic groups increases the membrane's affinity towards water molecules, reducing interfacial tension and enhancing the membrane's wetting properties. As a result, water molecules can penetrate and flow more easily through the membrane, leading to increased water flux and improved permeate flow. The highly interconnected pores and channels formed by the GO nanosheets provide more and more direct pathways for water molecules to travel through the membrane.^57,58^ Furthermore, the addition of GO nanoparticles to the membrane matrix improves its mechanical strength and stability, particularly under high *trans*-membrane pressures, enhancing the long-term performance of the membrane.^59^ Furthermore, incorporating GO into the membrane structure can enhance chlorine resistance due to its unique properties. GO acts as a barrier that effectively hinders the penetration of chlorine molecules into the membrane matrix and reduces its reactivity with –NH_2_ groups of MPD moieties of the membrane's PA layer.^60,61^ In another mechanism, the oxygen-containing functional groups of GO, such as hydroxyl and epoxide groups, provide preferential sites for chlorine molecules to react, where chlorine atoms can form covalent bonds with oxygen atoms on GO, resulting in chlorine–oxygen bonds that are more stable than chlorine–carbon bonds typically found in polymeric membrane material upon chlorination.^62,63^

Thus, the distinctive properties of GO nanosheets, including their hydrophilicity, chemical durability, and rapid water permeation, were used to develop a dual-function barrier coating for PA-TFC membranes.^29^ This conformal coating of GO effectively enhanced the membrane's surface hydrophilicity and reduced its roughness, leading to improved proteins' fouling resistance. Moreover, due to the chemically inert nature of GO nanosheets, the coating layer also served as a chlorine barrier, preventing membrane degradation when exposed to chlorine, while maintaining high salt rejection capability.^29^ On the other hand, reducing the polymer solution concentration used in the PA support layer preparation enhanced the TFC membrane water flux but resulted in a porous sub-surface structure that degraded the support layer's mechanical strength.^64^ Therefore, the researchers incorporated GO nanosheets into the sublayer, which enhanced the mechanical strength, porosity, and the water flux. These enhancements were affected by the GO nanosheets wt% and thickness, where higher content and thicker nanosheets had negative effects on the mechanical strength. The dispersion and surface area of the nanofillers also played important roles in their incorporation into the polymer matrix.

**Fig. 6:**
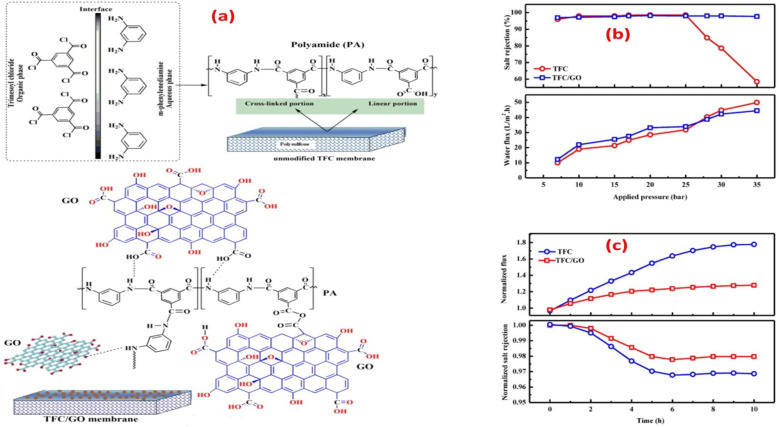
(a) Synthesis of PA with and without the addition of GO during interfacial polymerization; salt rejection and water flux of TFC and TFC/GO membranes at different (b) feed pressures, and (c) chlorine exposure time at a 500 ppm Cl_2_-dose. Reprinted with the permission of ref. 65, copyright 2024, Elsevier.

According to the findings, incorporating GO nanosheets with a thickness of 1.5 nm and 0.9 wt% dose yielded the maximum mechanical strength. The incorporation of GO did not affect the polyamide layer, as the active layer roughness and thickness were retained, and the network structure of the surface layer remained unchanged. Furthermore, the impact of incorporating GO during interfacial polymerization on membrane performance was examined, Fig. 6a.^65^ Namely, the membrane's water flux, salt rejection, and resistance to chlorine were assessed under various operating conditions, as shown in Fig. 6b and c, where TFC/GO membrane exhibited consistent salt rejection, whereas the pristine TFC membrane experienced a sharp decrease in salt rejection at high pressures due to deformation. Additionally, the TFC/GO showed enhanced chlorine resistance, Fig. 6c, as evidenced by higher normalized salt rejection and stable normalized flux, which can be attributed to the hydrogen bonding between the oxygen-bearing functional groups of GO and the secondary amide groups in PA, offering protection against chlorine. Furthermore, prior to interfacial polymerization, GO was added to the aqueous MPD solution to be embedded into the PA layer, and the resulting GO-TFN membranes showed 98% improvement in water anti-biofouling based on biovolume changes, as well as 80% increase in permeability features.^66^ These enhancements can be attributed to changes in surface charge, roughness, hydrophilicity, and PA layer thickness resulting from GO incorporation. In addition, the GO-TFN also exhibited a high salt rejection even when exposed to challenging conditions of 48 000 ppm h chlorination with a NaOCl solution, which can be attributed to the protection of PA-amide groups from chlorine attack. The study also emphasized the importance of GO concentration and size in influencing the performance of GO–TFN membranes.^66^ Furthermore, modification of RO membranes with GO improved surface properties, making them smoother and more hydrophilic, as well as reduced CaSO_4_ scaling and biofouling, demonstrating its potential for enhanced RO membrane performance.^67^ In another study, incorporating GO into the PA layer of TFC membrane had no effect on the membrane's permeability and salt rejection; however, it resulted in a noteworthy decline of viable *E. coli* cells, reducing them by 64.5% within one hour.^68^ GO induced membrane damage, mediated by physical disruption, charge transfer, formation of reactive oxygen species and lipid extraction from the cell membrane, as evidenced by SEM imaging, where most bacterial cells attached to the GO–TFC membrane appeared to be shrunk or flattened with compromised integrity compared to the control-TFC membrane.^68^

#### 2.1.2 Carbon nanotubes

Carbon nanotubes (CNTs) are made up of rolled-up cylindrical graphite sheets that resemble tubes and have the appearance of a mesh fence.^69^ CNTs exhibit superior thermal, mechanical, antibacterial and electrical properties. Because they can reduce the amount of energy required to remediate water, CNTs have intrigued researchers in the field of advanced membrane technologies for water desalination.^70,71^ CNT-modified membranes enable seamless water flow while effectively capturing a wide range of water pollutants. The hollow inner cavity of CNTs has substantial desalination potential. Notably, CNTs' unique properties, such as smooth hydrophobic walls, high aspect ratios, and small pore diameters, allow for remarkably appropriate water-molecules mobility; therefore, CNTs have received significant recognition in the field of RO membrane modification.^72^ The hydrophilicity and porous structure of CNTs, when introduced into TFC, can augment the water pathways within membranes and improve the affinity between membrane surface and water.^73^ Furthermore, the integration of negatively charged CNTs has the potential to raise the charge density of the membrane surface, lower the adhesion forces between the membrane surface and fouling agents, and thereby enhance its antifouling capabilities of the membrane. Studies have suggested that the stability of membranes against chlorine can be enhanced through the interactions between the AP-amide groups and the –COOH of CNTs.^74^ Additionally, CNTs possess inherent antimicrobial properties, which can help mitigate biofouling in RO membranes. The nanotube structure and surface chemistry of CNTs provide a hostile environment for the growth and adhesion of microorganisms, reducing biofilm formation and microbial fouling.^75^ Furthermore, the introduction of CNTs into the membrane composite may yield a synergistic effect, leading to an enhancement in the photocatalytic features of the membrane.^76^ CNTs can provide mechanical reinforcement to the membrane structure, enhancing its stability and resistance to physical stress.^75^ The robust nature of CNTs helps to prevent the membrane from deformation or damage during operation, leading to improved membrane performance and lifespan.

For instance, incorporating MWCNTs into the PA layer of a TFC membrane resulted in enhanced chlorine resistance that was attributed to the electron-rich MWCNTs protection of the amide linkage.^77^ Furthermore, TMC solutions in *n*-hexane were mixed with MPD solutions containing modified CNTs to prepare PA thin films *via* controlled interfacial polymerization (IP) process, resulting in the formation of well-dispersed states.^78^ In that work, CNTs were previously treated for several hours (*x*) at predetermined settings with various mixtures of sulphuric and nitric acids to impart oxygen bearing functionalities to the modified CNT*x*. Among the prepared membranes, PA-CNT4 demonstrated enhanced durability, water flux, and chemical resistance compared to the pristine PA membranes. These improved performance features were attributed to the presence of hydrophobic nanochannels provided by the CNTs. Additionally, incorporating CNTs into PA films of TFC membranes improved water permeability, leading to reduced energy consumption, high anti-fouling potential, and improved recovery efficiency during the cleaning stage due to the smoother surfaces.^79^ Furthermore, the effect of varying amounts of CNTs on the performance of PA membranes was examined.^75^ The experimental findings demonstrated promising results, with notable improvements observed in salt rejection, high water flux, and remarkable anti-biofouling properties, as well as enhanced durability. However, when higher amounts of CNTs were incorporated, the anti-biofouling characteristics of the PA–CNT membrane coated with polyvinyl alcohol (PVA) were reduced. This decline in anti-biofouling efficacy was attributed to the formation of aggregated CNT clusters within the membrane. The superior anti-biofouling properties observed in the PA–CNT–PVA membrane were primarily attributed to the inherent antimicrobial properties of the CNTs. To validate this, both confocal laser scanning microscopy (CLSM) imaging and cell viability tests were conducted, providing evidence of the membrane's anti-biofouling efficacy.^75^

**Fig. 7:**
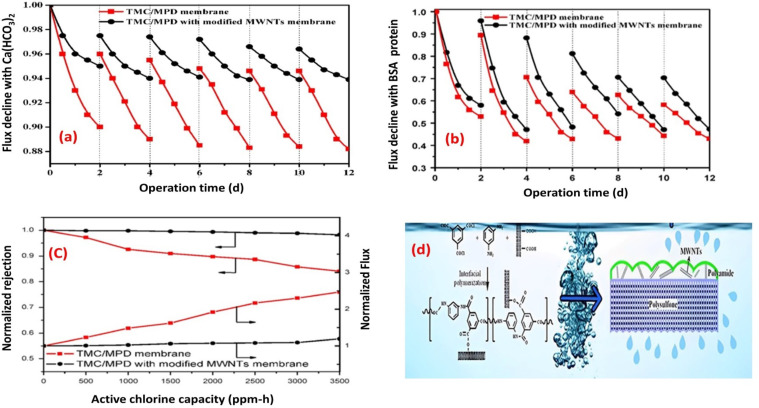
Flux decline for TMC/MPD and TMC/MPD/MWNTs membranes due to (a) Ca(HCO_3_)_2_, (b) BSA-protein foulant, (c) RO performance under the active chlorine, and (d) schematic fabrication of TMC/MPD/MWNTs membrane. Reprinted with the permission of ref. 73, copyright 2024, Elsevier.

Layer to enhance its resistance to fouling and chorine as well.^73^ Experimental tests conducted using inorganic (Ca(HCO_3_)_2_) and BSA-protein foulants demonstrated that the TFN membrane experienced a lower decline in water flux and exhibited reduced irreversible fouling, as shown in Fig. 7a and b, respectively. These improvements were primarily attributed to the enhanced electrostatic repulsion between the foulants and the membrane surface, as well as the improved hydrophilicity of the TFN membrane, which minimized foulant adhesion. Furthermore, the TFN membrane also exhibited high resistance to chlorine, which can be attributed to the MWCNTs electron-rich nature that provide protection to MPD active sites, and hence hindering the chlorine attack (Fig. 7c and d).^73^ Furthermore, incorporation of raw and oxidized MWCNTs reduced inorganic-, organic- and bio-fouling owing to the increased membrane's negative charge and smooth surfaces.^80^ Additionally, the fabricated membrane exhibited twice the lifespan in comparison to existing commercially available membranes.^80^ Results reflected that the hydrophilicity, as well as water flux, improved with increasing concentration of oxidized MWCNTs compared to bare PA or raw MWCNTs/PA. Despite their lower hydrophilicity, the membranes with raw MWCNTs exhibited significant improvements in water flux and salt rejection due to their proper dispersion within the polyamide matrix. However, at higher concentrations of both MWCNT types, the water flux decreased. Assessment of fouling behavior in a 24 hours test using BSA/salt solution demonstrated that membranes with all concentrations of MWCNTs, whether raw or oxidized, exhibited better antifouling performance compared to unmodified membranes. Finally, optimized concentrations played a crucial role in achieving superior membrane characteristics.^80^

In another investigation,^81^ the researchers follow the efficiently reduced protein adhesion and antifouling capabilities of MWCNT–PA by employing a multifaceted approach combining molecular dynamics (MD) simulations and experimental studies. MD simulations, suggested that the incorporation of MWCNTs within the PA framework gives rise to weaker interactions between BSA proteins and membrane surfaces. This weakened interaction can be attributed to three key factors: a more rigid PA structure resulting in reduced molecular conformity with BSA, enhanced hydrophilicity, and smoother morphology leading to the formation of an interfacial water layer.^81^ Similarly, a fabricated MWCNTs–PA exhibited resistance to chlorine attacks that as ascribed to the electronic-rich nature of the highly sp^2^-hybridized MWCNTs and the existence abundance of sacrificial organic functionalities on nanotubes. Moreover, the physical crosslinking effect of MWCNT also contributes to the membrane's resistance by preventing the formation of pinholes. It should be noted that PA with a significant degree of crosslinking is generally more resistant to chlorine attack.^82^

#### 2.1.3 Metal–organic frameworks (MOF)

MOFs are porous materials consisting of metal ions or clusters connected by organic ligands. Their unique structure and tunable properties make them versatile materials for addressing environmental challenges. MOFs typically exhibit exceptionally significant surface areas, often surpassing 1000 to 7000 m^2^ g^−1^.^83^ The structure of MOFs can be specifically tailored to have different pore sizes, shapes, and functionalities.^84^ Moreover, many MOFs possess inherent catalytic activity, either due to the metal centers or the organic linkers in their structure.^85,86^ They possess the flexibility of organic matter and the stiffness of porous inorganic material.^87,88^

The enhanced performance of reverse osmosis (RO) membranes upon incorporating metal–organic framework (MOF) materials can be attributed to several key mechanisms. Firstly, MOFs can provide a high density of active sites for selective interaction with water molecules, leading to improved water permeability. The porous structure of MOFs allows for the formation of nanoscale channels, increasing the overall water flux through the membrane.^89,90^ The permeation characteristics of a MOF-TFN are significantly altered by the size of the pores of a MOF.^89^ Furthermore, MOFs can enhance the salt rejection capabilities of RO membranes. Additionally, by functionalizing the MOF materials with specific functional groups or modifying the ligands, the selectivity towards certain ions can be increased.

For instance, this selectivity can be especially valuable in applications like seawater desalination, where high salt rejection rates are crucial for obtaining fresh water. Thus, the addition of myristic acid (MA) to zirconium-based porphyrinic MFOs (PCN-222) reduces pore size and results in improved rejection compared to pristine MOFs.^89^ Additionally, MOFs can enhance the stability and durability of RO membranes. The incorporation of MOFs into the polymeric matrix of the membrane can improve its mechanical strength and resistance to fouling.^78,79^ Moreover, the high thermal stability of MOFs allows for their application in harsh operating conditions, such as high temperatures or corrosive environments. This expands the range of potential applications for RO membranes, making them suitable for a broader range of industries and processes.

**Fig. 8:**
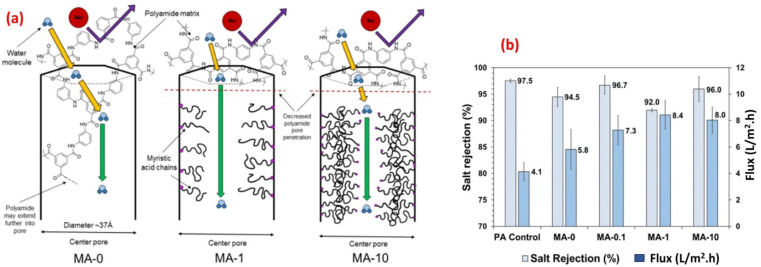
(a) Schematics of water transport through the pores of zirconium-based porphyrinic MOFs (PCN-222 nanorods) with increasing modifier loading (myristic acid, MA, at 0, 0.1, 1 and 10%), and (b) salt rejection and water flux results for MA-modified and pristine membrane reprinted with the permission of ref. 89, copyright 2024, American Chemical Society.

Furthermore, the researchers examined the potential of a microporous and hydrophobic hybrid zeolitic imidazole frameworks substance (ZIF-8) as filler in TFC-RO membranes. They observed a remarkable enhancement in water permeance of up to 162% while keeping an impressive NaCl rejection rate of 98%. Additionally, the selective layer's surface, when integrated with ZIF-8, exhibited more hydrophilicity and reduced crosslinking compared to the pristine polyamide (PA).^79,91^ Additionally, to enhance the water permeability, and the support layer's porosity and hydrophilicity, researchers developed a polysulfone (PSf) layer incorporating a sulfuric acid-treated HKUST-1 MOF, [Cu_3_(1,3,5-benzenetricarboxylate)_2_·(H_2_O)_3_]_*n*_.^91,92^ The overall surface roughness of the TFC was affected by the support layer's porosity and hydrophilicity. By improving these features in the support layer, they successfully produced a TFC-RO membrane with reduced roughness which resulted in enhanced resistance to fouling and improved water flux compared to a TFC-RO with a bare PSf, whilst maintaining its excellent salt rejection capability.^93^ The rodlike structure of PCN-222 MOFs has been successfully fabricated and incorporated into TFC RO.^89^ Furthermore, the PCN-222 was modified with MA after synthesis, allowing for the adjustment of the MOF pore size (Fig. 8a). Membranes fabricated using both bare and modified PCN-222 MOF demonstrated slight reductions in selectivity and significant increase in water flux compared to bare PA membranes. Specifically, the modified PCN-222 -TNF membranes (MA-10) exhibited an almost 100% enhancement in flux while maintaining salt rejection above 95% (Fig. 8b).^89^ The authors proposed that the existence of hydrophobic MA chains hinders the infiltration of MPD into the pores of the PCN-222 nanoparticles in suspension, leading to a reduction in pore penetration by the polyamide (Fig. 8a). This causes an increase in pore volume available for transport, resulting in higher flux and lower salt rejection compared to the control membrane (Fig. 8b). Additional mechanisms that could contribute to the enhanced flux in the PA/PCN-222 include interfacial movement occurring at the surface of the MOF aggregates, as well as transport taking place within the interstitial channels of the MOF aggregates.^89^

In another investigation, a microporous and hydrophilic hybrid MOFs, MIL-101 (Cr), was incorporated into a TCF RO membrane, resulting in improved overall membrane performance.^90^ This enhanced performance can be attributed to changes in membrane characteristics such as roughness, crosslinking extent, morphology, and wettability. Moreover, doped MIL-101 (Cr) also creates direct channels within the PA layer of the membrane. At a concentration of 0.05 w/v% of MIL-101 (Cr), water permeability increased by up to 44% while keeping NaCl salt rejection over 99.^91^ Overall, the integration of metal–organic framework materials into RO membranes can significantly enhance their performance. MOFs offer improved water permeability, higher salt rejection rates, enhanced stability, and increased durability.

### 2.2 Incorporation of inorganic nanomaterials

The inclusion of inorganic nanomaterials into the polymer matrix has gained attention due to their hydrophilic characteristics, which can enhance a range of physical and biomedical benefits, such as optical, mechanical, catalytic, and antimicrobial membrane properties.^94,95^ Through the addition of inorganic NPs, the permeability of pure polyamide membranes can be enhanced by facilitating water transport through a direct pathway or modifying the membrane structure.^21^ Consequently, the development of PA-TFC reverse osmosis membranes incorporating inorganic nanomaterials like pure zeolites, metal oxides, and metal nanoparticles has resulted in improved separation performance.^96–98^Table 2. Shows the performance evaluation of several TFN reverse osmosis membranes with incorporation various inorganic-nanoparticle.

**Table 2:** Performance evaluation of several TFN reverse osmosis membranes incorporating various inorganic nanomaterials

Modifier type	Test conditions TDS (ppm), temperature, pressure	Flux	Rejection %	Remarks	Ref.
Mesoporous SiO_2_ NPs	2000, NA, 16 bar	19 to 53.0	>96	Mesoporous silica produces hydrophilic pores in RO membranes' thin film layer, enhancing water diffusion and improving water flux	155
TiO_2_ NPs	2000, NA, 15.5 bar	33.6 to 40	99.7 to 99.8	Low-concentration TiO_2_ in the synthesized TFNC membrane improved its resistance to organic fouling	136
TiO_2_ NPs	2000, 25 °C, 15.2 bar	21.5 to 24.3	>97	The thermal stability and anti-biofouling of TFN membranes were enhanced by the incorporation of TiO_2_	50
Fluorinated silica	2000, 25 °C, 15.5 bar	39.3	96.0 to 98	Incorporation of hydrophobic fluorinated silica increasing membrane hydrophobicity and decreased salt passage	156
Pours SiO_2_	2000, 25 °C, 20.6 bar	28.5 to 46.6	98 to 97.5	Interior pores, due to their narrow flow routes, significantly enhance water flux, as evidenced by the disparities in water flux between two membranes	157
Non porous SiO_2_	2000, 25 °C, 20.6 bar	28.5 to 35.8	98.0 to 97.0		
rGO/TiO_2_	2000, NA, 15.0 bar	34.3 to 51.3	97 to 99.45	The addition of rGO/TiO_2_ improved the hydrophilicity, negative surface charge, and roughness of the polyamide layer of the RO membrane, which improved water permeability, salt rejection, antifouling, and chlorine resistance	29
				Due to TiO_2_ nanoparticles' increased affinity for water, the rGO/TiO_2_/RO membranes' increased hydrophilicity may be the cause of this improved permeability	
SiO_2_	11 000, NA, 44 bar	31 to 49	>90.0	The integration of SiO_2_ nanoparticles reduces the fouling tendency of the polyamide active thin layer, making it acceptable due to its hydrophilic properties	30
CeO_2_	2000, NA, 16 bar	29.4 to 49.3	≥98	By limiting the adherence of hydrophobic foulants, CeO_2_ embedded membranes demonstrated improved fouling resistance	158
Aluminum doped ZnO	2000, 25 °C, 15.5 bar	26 to 32	>98.0	The structure and hydrophilicity of membranes, influenced by hydrophilic nano-ZnO, significantly influence their filtration properties, leading to higher water flux in hybrid membranes compared to TFC membranes	147
AgNPs	2000, NA, 20 bar	NA	95 to −98	The enhancement in rejection can be assigned to Donnan and size exclusion mechanism	147
NaY zeolite	2000, NA, 15.5 bar	39.6 to 74.2	98.5 to 98.8	The incorporation of NaY zeolite exhibits a molecular sieve effect, allowing water molecules to pass preferentially while rejecting solute molecules, due to their homogeneous porosity structure and 0.74 nm pore size	55

#### 2.2.1 Zeolite nanoparticles

Zeolites are crystalline aluminosilicate materials that possess unique characteristics, making them highly valuable in various fields, including catalysis, adsorption, and ion exchange.^99–101^ These minerals have a three-dimensional porous structure with a regular arrangement of channels and cavities. With a porous structure composed of interconnected silicon and aluminum tetrahedra bridged by oxygen atoms,^101^ zeolites offer a high surface area and tunable pore size and shape, leading to efficient molecule and ion interactions.^102,103^ Their ion exchange capabilities enable the selective removal of specific ions and pollutants. Moreover, zeolites exhibit exceptional thermal and chemical stability, making them suitable for harsh conditions.^102^ These unique characteristics have generated interest in incorporating zeolite nanoparticles into systems such as membranes separation processes. The presence of zeolite creates additional pathways for water molecules to pass through, promoting faster and more efficient water transport. This increased permeability results in higher water flux rates and improved overall membrane performance.^104^

When RO is modified with zeolite, the enhanced performance can be attributed to several mechanisms. The negatively charged molecular sieve and super-hydrophilic zeolite will facilitate the selective movement of water by establishing specific pathways while giving effective solute rejection by utilizing a combination of Donnan and steric exclusion mechanisms.^96^ Zeolites contain pores with sizes ranging from approximately 0.3–0.8 nm. These pore sizes are positioned between the diameters of hydrated salt ions, such as Na^+^ (0.72 nm), and water molecules (0.27 nm).^104^ Taking advantage of the principle of size exclusion, zeolites are expected to facilitate the preferential permeation of water while impeding the passage of salt ions.

For instance a 12–16% improvement in water flow with zeolite-TFN sea water reverse osmosis (SWRO) membranes was observed compared to unmodified SWRO membranes with similar salt removal rates.^105^ The researchers attributed this enhancement to both molecular sieving and the formation of defects in the TFN-SWRO membranes, as well as the low degree of crosslinking due to the presence of zeolite despite of its low concentration (0.2 wt%).^105^ Further, incorporating sodium-aluminum-silicate zeolite (NaA-NPs) into the active PA layer of TFC-RO membranes resulted in more hydrophilic, smoother, and more negatively charged membrane surfaces owing to the characteristics of NaA zeolite which results in improved salt rejection and enhanced antifouling features.^96^ The highest zeolite loading content (0.5%) led to a reduction in contact angle from about 70° to 40° and increased permeability while maintaining solute rejection. This improvement can be attributed to the preferential flow paths for water permeation provided by NaA zeolite, along with steric and Donnan exclusion mechanisms.^96^

Additionally, two types of zeolite, hydrophilic (FAU, Al/Si = 1.0) and hydrophobic (MFI, Al/Si = 0), were incorporated into RO membranes.^106^ Both FAU and MFI membranes demonstrated a remarkable salt rejection rate of 100%, with improved membrane permeability reaching 720 L m^−2^ h^−1^ bar^−1^ for a membrane thickness below 3.5 nm, that is approximately 100 times higher than the permeability of commercial RO membranes.^106^ Additionally, the FAU membrane showed a lower pressure drop due to its hydrophilic nature, while the hydrophobic MFI zeolite membrane displayed a higher pressure drop attributed to capillary resistance.^106^

In addition, nonthermal glow discharge plasma was used to incorporate clinoptilolite zeolite into the polyamide (PA) layer of the TFC-RO membrane and the resulting composite membrane demonstrated significant enhancement in water flux and fouling recovery ratio when compared to the unmodified membrane.^20^ Moreover, the researchers investigated the impacts of incorporating nano-NaX zeolite into PA membranes, and observed several improvements in the surface features of the nanocomposite.^107^ These enhancements included a reduction in root mean square (RMS) surface roughness, an increase in free energy of liquid–solid interface, and a decrease in contact angle. Furthermore, the addition of nano-NaX zeolite resulted in larger pore size, a thinner film thickness, higher thermal stability, and improved water flux compared to pure PA membranes.^107^ Additionally, TFN membranes integrated with NaY zeolite nanoparticles were successfully fabricated on polysulfone supports through interfacial polymerization of MPD and TMC.^108^ The experimental findings revealed that increasing the reaction time during IP process resulted in higher salt rejection, indicating a denser zeolite-polyamide layer, along with enhancing the water flux from 39.63 to 74.32 L m^−2^ h^−1^, while maintaining a salt rejection of 98.8% under optimized conditions. Through post-treatment of the TFN membranes using a solution composed of camphor sulfonic acid-triethylamine salt, sodium lauryl sulfate, and glycerol, the water flux was further enhanced to 86.05 L m^−2^ h^−1^, with a salt rejection of 98.4%. Comparatively, the water flux of the post-treated TFN membranes containing NaY zeolite nanoparticles was twice as high as that of a pristine TFC membrane without zeolite nanoparticles.^108^

#### 2.2.2 Silica nanoparticles

Silica, also known as silicon dioxide (SiO_2_), is a naturally occurring compound that is one of the most abundant minerals on Earth. Silica possesses unique physical–chemical properties that make it a versatile and valuable material. It is chemically inert, which means that it does not react with most substances, making it an excellent choice for applications where chemical stability is crucial. Mesoporous silica is a potential material in many fields due to its unique properties such as large surface area, mesopore homogeneity, surface hydrophilicity with many silicon hydroxyl groups, and controlled nanostructures and macroscopic morphologies.^109–111^ Silica or mesoporous silica nanoparticles are known as hollow-based nanoparticles that have an appropriate size with a large pore volume and surface area. Silica nanoparticles have pores that range from 2–20 nm in size, which give them a large surface area (>1000 m^2^ g^−1^), excellent pore volume, and superior chemical stability.^112,113^ Pristine silica membranes can form microporous structures, but they tend to become unstable during the process of desalination. This is because the water that passes through the micropores interacts with silane groups, causing the silica film's pore sizes to increase.^114^ To enhance both the mechanical and chemical stability of these membranes, it may be necessary to incorporate silica nanoparticles into polymeric nanocomposites.^114^ Silica's incorporation into RO membranes provides several benefits, including improved permeability efficiency, fouling resistance, and overall system durability. Silica promotes the formation of a more hydrophilic membrane surface which enhances the water flux through the membrane by reducing the membrane's resistance to water flow.^115^ Furthermore, silica incorporation enhances the durability of the RO membrane by reducing membrane degradation. Silica particles act as buffering agents, absorbing, and dissipating thermal, mechanical stresses and pressure fluctuations within the RO system.^116–118^

**Fig. 9:**
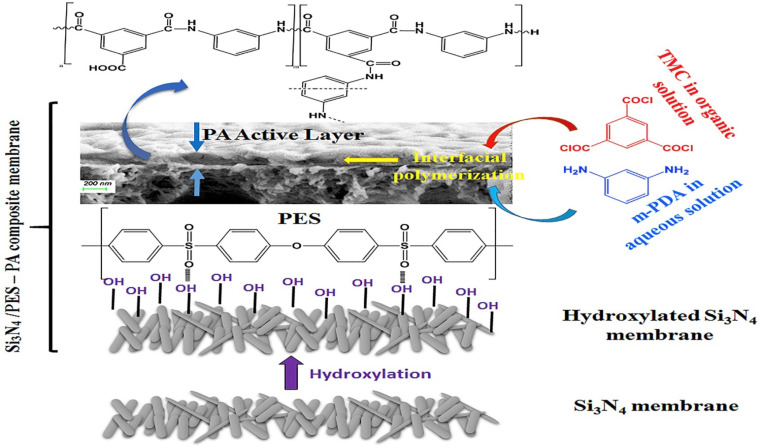
TFC synthesis based on Si_3_N_4_/polyethersulfone (PES) composite membrane. Reprinted with the permission of ref. 118, copyright 2024, Elsevier.

For instance, the researchers examined the utilization of SiO_2_ NPs as nanofillers in PA membranes to improve their performance. They created TFN membranes using SiO_2_ NPs of various sizes (10–20 nm) and ratios. The inclusion of SiO_2_ NPs led to structural modifications in the membranes, with higher concentrations of SiO_2_ resulting in a more extensive porous network. The incorporation of SiO_2_ NPs led to a considerable improvement in permeability, increasing it by 58%. When compared to the pristine membrane, the modified membranes with SiO_2_ demonstrated higher permeability and lower flux decline ratio, while filtering organic matter solutions. This investigation offers valuable insights into the potential of SiO_2_ NPs as a viable strategy for enhancing the performance of PA membranes.^119^ For instance, the incorporation of SiO_2_ NPs into RO membranes led to enhanced thermal stability, as indicated by a minimal decrease in salt rejection of less than 2.5% after heat treatment at 95 °C for 180 minutes.^120^ Furthermore, the membranes demonstrated excellent resistance to chlorine exposure, with a salt rejection decrease of less than 2% even after being exposed to 10 000 ppm h of NaOCl.^120^ Further, TFN membranes were fabricated by IP process of MPD and TMC organic solution on silicon nitride/polyethersulfone (PES) composite substrate, as shown in Fig. 9.^118^ Researchers have found that by introducing amino functionalization to silica (SNPs) using *p*-aminophenol, the compatibility of silica NPs with the PA layer can be enhanced. This modification also reduces the occurrence of agglomeration during the interfacial polymerization process, thereby facilitating the successful production of high-quality TFN membranes. The TFN shows considerably improved flux, compared to the pristine TFC membrane, along with higher thermal stability.^118^

#### 2.2.3 TiO_2_ nanoparticles

**Fig. 10:**
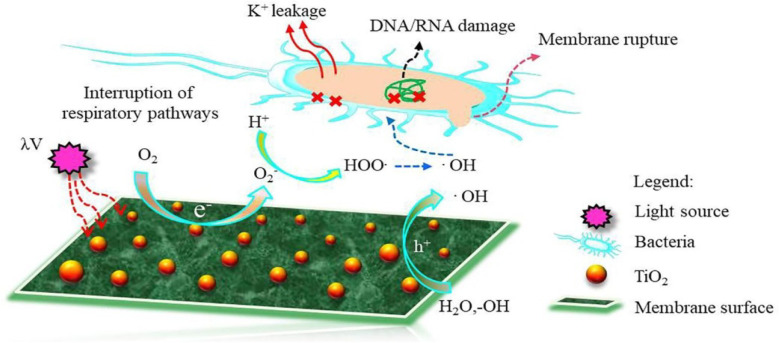
Proposed mechanism of ROS formation on the surface of TiO_2_ NPs-modified TFC membrane. Reprinted with the permission of ref. 135, copyright 2024, Elsevier.

The modification of RO membranes with titanium dioxide (TiO_2_) has gained significant attention due to its potential to enhance membrane performance.^121^ Several mechanisms contribute to the improved performance of TiO_2_-modified RO membranes. One key mechanism is the photocatalytic activity of TiO_2_ nanoparticles. When irradiated with UV light, TiO_2_ nanoparticles generate reactive oxygen species (ROS) that can degrade organic compounds and microbial pollutants present in the water.^49,122^ This photocatalytic effect helps to mitigate bacterial and biofouling issues, leading to improved membrane performance and reduced maintenance requirements (Fig. 10).^121,123–125^ Another mechanism is the hydrophilicity enhancement of TiO_2_-modified membranes. TiO_2_ nanoparticles possess a high surface area and can alter the surface properties of the membrane, making it more hydrophilic. This hydrophilic surface reduces fouling by inhibiting the adsorption of organic matter and preventing the attachment of foulants, thereby enhancing the water permeability and flux of the membrane.^126^ Additionally, TiO_2_ can provide structural stability to the RO membrane.

For instance, PA/TiO_2_ TFN membranes were synthesized using an *in situ* interfacial polymerization,^126^ where two different approaches were utilized to incorporate TiO_2_ nanoparticles into the membranes: dispersing them in the dodecane organic phase of TMC or the aqueous phase of MPD. The synthesized PA–TiO_2_ TFN membranes exhibited a significant enhancement in the permeate flux that increased from 33.6 to 40 L m^−2^ h^−1^, indicating improved water productivity. Additionally, there was a slight increase in salt rejection, rising from 99.75% to 99.82%. Moreover, the TFN membrane with a lower TiO_2_ concentration demonstrated improved resistance to organic fouling.^126^

**Fig. 11:**
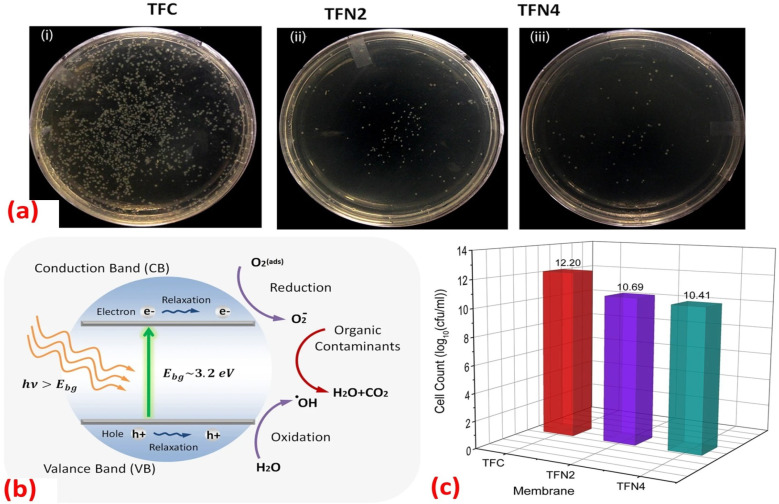
(a) Images of the *E. coli* colonies formed in the plate of UV-treated (i) TFC, (ii) TFN2 (0.012 wt% TiO_2_) and (iii) TFN4 (0.024 wt% TiO_2_) membranes; (b) mechanism for photocatalytic activity of TiO_2_ NPs under UV irradiation; (c) number of *E. coli* colonies counted on the plate of TFC, TFN2 and TFN4 membranes after 30 minutes of UV irradiation.^127^

Furthermore, the impact of incorporating TiO_2_ NPs, at different NP concentrations (0.01, 0.05, 0.2, and 0.5% w/v) into PA on the rejection of organic matter (OM) and salts as well as permeate flux, was assessed.^119^ The inclusion of TiO_2_ nanofillers in the PA membranes enhances their porosity, hydrophilicity, and consequently, their permeability that increased by 24% compared to membranes without NPs, where the rejection performance and fouling behavior of the membranes were assessed using salts (MgSO_4_ and NaCl) and OM (humic acid [HA] and tannic acid [TA]). In addition, sub-10 nm TiO_2_ NPs were incorporated, at different doses, into the PA layer to create TFN.^127^ The researchers found that these TFN membranes exhibited enhanced thermal stability and antifouling properties compared to pristine membranes. The number of *E. coli* colonies that formed over the UV-illuminated membranes was counted, Fig. 11. The results revealed that the exposure of TiO_2_-incorporated TFN membranes to UV light significantly reduced the viability of *E. coli*.^127^ However, despite its effectiveness, the use of TiO_2_-based antifouling membranes is hindered by the requirement of costly UV irradiation.^123^ This has limited their practical application.^123^ However, there has been considerable research directed toward the development of visible-light responsive-TiO_2_ that utilizes the localized surface plasmon resonance (LSPR) of metal nanoparticles, specifically gold (Au) and silver (Ag) nanoparticles.^123,128^

Overall, the enhanced performance of RO membranes modified with TiO_2_ is driven by its photocatalytic activity, improved hydrophilicity, antimicrobial properties, and structural reinforcement. These mechanisms collectively enable the membrane to resist fouling, prevent microbial growth, increase water permeability, and extend its lifespan.

#### 2.2.4 Silver NPs-modified TFC membranes

**Fig. 12:**
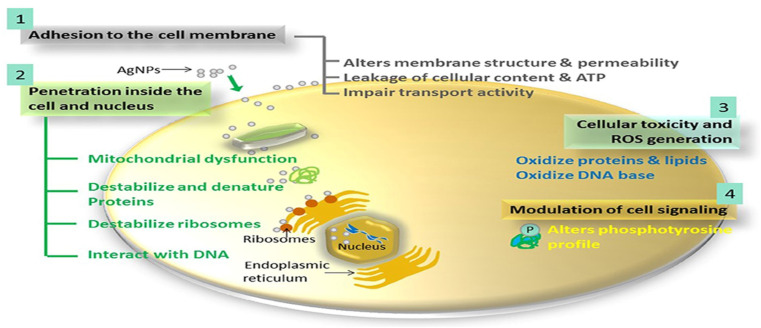
The proposed antimicrobial mechanisms of Ag NPs.^130^

Silver NPs have proven to be effective in enhancing the performance of RO membranes, preventing biofouling, improving thermal response, facilitating photocatalytic degradation, and enhancing electro-conductivity.^129^ Ag-NPs have widely been recognized for their antibacterial properties, which occur through various mechanisms, including the generation of reactive oxygen species (ROS), contact killing, and the release of silver ions (Ag^+^) (Fig. 12).^130^ To further elaborate, Ag-NPs have a strong affinity for the cell membrane surface, allowing them to penetrate into the cell and disrupt its functions. Furthermore, the production of ROS by Ag-NPs leads to damage to the cell membrane and the alteration of its DNA structure. The release of Ag^+^ ions can disrupt the production of adenosine triphosphate (ATP) and hinder DNA replication within the cell membrane.^131,132^ Additionally, the application of the Schottky effect^133^ and surface plasmon resonance (SPR)^134^ can enhance the absorption of visible light by photocatalytic materials when combined with Ag-NPs. These improvements in photocatalytic performance can enhance the antibacterial and organic degradation capabilities of modified membranes. Furthermore, Ag-NPs possess strong biocidal properties, and the increased hydrophilicity of the Ag-TFC membrane can help prevent bacterial adhesion, thereby reducing the likelihood of biofouling.^135–137^

**Fig. 13:**
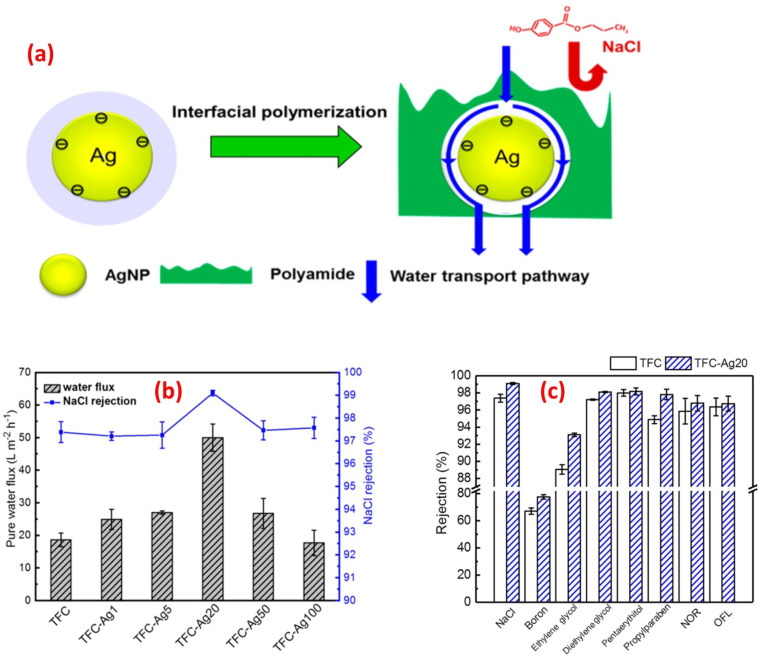
(a) Schematic diagram of Ag-NPs induced nanochannels in the polyamide layer for efficient water transport, (b) separation performance results of the control and TFC-Ag20 membranes, and (c) membrane rejection results of the control TFC and TFC-Ag20 membranes (the suffix Ag20 means prepared using 20 mM solution of AgNO_3_). Reprinted with the permission of ref. 147, copyright 2024, American Chemical Society.

For instance, the antibacterial characteristics of a covalently bonded cysteamine-modified TFC membrane incorporating Ag-NPs was investigated.^138^ Compared to the pristine TFC membrane, the Ag-NPs-grafted TFC membrane exhibited slightly lower salt rejection but a higher water flux and potent antibacterial properties against *E. coli*, evidenced by the absence of bacterial growth on the membrane surface. The antimicrobial mechanism of Ag-NPs can be attributed to their ability to disrupt bacterial cell metabolism. The antimicrobial effect of Ag^+^ ions involves the inactivation of membrane proteins followed by binding to bacterial DNA, which interferes with DNA replication.^138^ The facile loading of Ag-NPs onto TFC-RO membranes was achieved through the reaction of a reducing agent with a silver salt on the surface of the membrane to yield a uniform Ag-NPs coverage with firm bonding to the membrane.^97^ These Ag-NPs demonstrated strong antibacterial activity, resulting in a decrease of over 75% in the number of viable bacteria attached to the membrane for different bacterial strains. Additionally, using confocal microscopy, the researchers observed that Ag-NPs effectively inhibited biofilm formation, leading to a significant decrease in total biomass, as well as reduced levels of EPS and both live and dead bacteria on the membrane.^97^ Furthermore, a study of Ag-NPs TFN membrane unveils the creation of nanochannels with an average size of approximately 2.5 nm around the Ag-NPs.^139^ The formation of these nanochannels can be attributed to the hydrolysis of TMC monomers and the subsequent termination of IP by the water layer surrounding each hydrophilic Ag-NPs, as shown in Fig. 13a. Importantly, these nanochannels significantly enhanced the water permeability of TFN membranes, nearly tripling it compared to the pristine membrane without nanochannels, Fig. 13b. Furthermore, the incorporation of Ag-NPs leads to improved rejection capabilities against various substances such as NaCl, boron, and small organic compounds like norfloxacin, propylparaben, and ofloxacin, Fig. 13. This enhanced rejection performance can be attributed to a combination of factors, including enhanced Donnan exclusion, improved size exclusion, and suppressed hydrophobic interaction.^139^

## 3. Characterization of polyamide layers

Characterization techniques play a vital role in studying the physicochemical properties of nanoparticles incorporated into/onto thin film composite (TFC) membranes.^140^ These techniques enable researchers to acquire valuable information about the morphology, composition, and interactions between nanoparticles and membrane surfaces. This overview section presents a classification of characterization techniques, highlighting their types and importance in providing essential information for assessing the performance of nanoparticles-modified membranes.

### 3.1 Surface imaging techniques

Surface imaging techniques, such as atomic force microscopy (AFM), and scanning and tunneling electron microscopy (SEM, and TEM) are of significant importance in characterizing nanoparticles-modified RO membranes.^141,142^ These techniques provide valuable insights into the surface morphology, roughness, thickness, and distribution of nanoparticles on the membrane surface. This information is critical for understanding the interaction between nanoparticles and the membrane surface, assessing the uniformity and coverage of the modification layer, and evaluating the potential impact on membrane performance.

**Fig. 14:**
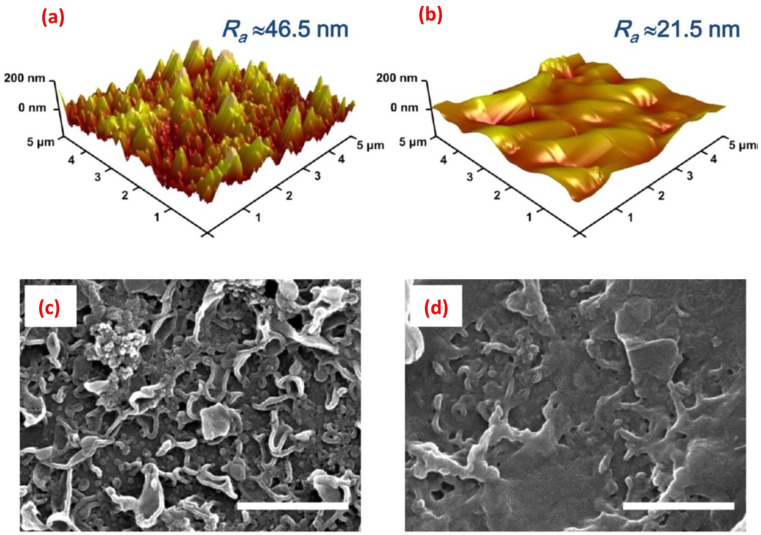
AFM height images of (a) the pristine, uncoated polyamide (PA) and (b) the GO_10_-coated PA membranes; and the SEM images of (c) the pristine PA and (d) the GO_10_-coated PA membranes, (scale bar = 1 μm). Reprinted with the permission of ref. 29, copyright 2024, American Chemical Society.

AFM enables high-resolution imaging of the membrane surface, which allows for the assessment of nanoparticle adhesion, distribution, and stability. It provides information about the surface topography, roughness, and porosity, aiding in understanding the surface modifications caused by the incorporated nanoparticles. AFM offers detailed insights into the surface features at the nanoscale level, which are crucial for evaluating the impact of nanoparticle modifications on membrane performance. Additionally, AFM can measure the thickness of the nanoparticle layer, further assisting in optimizing the coating process. For instance, AFM analysis revealed that the PA membrane coated with 10% w/w graphene oxide (GO10-PA), displayed an average roughness (*R*_a_) of approximately 21.5 nm. This value was significantly lower compared to the pristine PA membrane, which exhibited an *R*_a_ of approximately 46.5 nm (Fig. 14a and b).^29^ In another investigation, AFM images provided evidence showcasing that the surface roughness of membrane increased proportionally with the concentration of SiO_2_.^31^ Further, AFM analysis of the (PA/GO) composite revealed non-uniform coverage of GO NPs, with some areas showing folded GO NPs. Additionally, the surface roughness exhibited slight variations across different regions of the sample. These observations can be attributed to the uneven distribution of GO NPs within the PA matrix. Furthermore, due to the thickness of GO NPs ranging from 1.1 to 2.6 nm, the incorporation of GO NPs resulted in a slightly thicker membrane formation.^60^ In another study,^62^ it was observed that the surface modification of PA resulted in a smoother surface, evidenced by a decrease in roughness (*R*_a_) from 66.4 nm to 50.3 nm. Similarly, AFM analysis showed that as the content of the reduced graphene oxide (rGO)-TiO_2_ nanocomposite increased, the TFN-RO membrane roughness gradually decreased.^30^

SEM offers high-resolution imaging of the modified membrane surface, enabling researchers to scrutinize nanoscale features and assess the distribution of nanoparticles. It provides a larger field of view compared to AFM, allowing for the examination of a larger sample area. With SEM, the morphology, cross-sectional views, shape, and spatial arrangement of nanoparticles on the membrane surface can be observed, aiding in understanding the efficacy of the modification process.^143,144^ Thus, SEM was employed to examine the surface structure of SiO_2_-modified TFC membrane.^145^ The researchers observed a nodular pattern on the membrane surface, consisting of densely packed granular bumps, where the addition of SiO_2_ nanoparticles resulted in the formation of prominent ridge-like nanoribbons, clearly visible on the surface of the modified membrane. As the concentration of the added SiO_2_ NPs increased, the substrate surface exhibited an enhanced presence of these distinct structural characteristics, resembling a nanoribbon polyamide (PA) layer.^145^ In another study, SEM images revealed the existence of spherical 10–20 nm sized Ag-NPs on the membrane surface.^138^ Additionally, SEM images of the original PA membrane surface displayed a rugged topography characterized by ridges and valleys; however, when planar aminated graphene oxide (AGO) and GO nanosheets are introduced, they effectively smoothened the surface by filling in the valleys (Fig. 14c and d).^29^ Further, by utilizing SEM analysis, researchers could obtain high-resolution images of the layers cross-section, revealing important information about the layer-by-layer structure, thickness, and spatial arrangement of the nanomaterials in the modified RO membranes. Therefore, to examine the PA layer's cross-section, membrane samples are prepared by either removing the polyester backing layer and fracturing the polyamide support layers, or immersing the entire membrane in liquid nitrogen and then fracturing the polyamide support layer.^146^ Other studies have used SEM analysis to investigate the cross-section of TFN membranes.^66,147–149^

Additionally, SEM can be combined with energy-dispersive X-ray spectroscopy (EDS) to obtain elemental composition information, helping to identify the presence and distribution of nanoparticles on the membrane surface.^127,141^

### 3.2 Chemical composition analysis

Chemical composition analysis techniques, such as X-ray Photoelectron Spectroscopy (XPS) and Fourier Transform Infrared Spectroscopy (FTIR), provide in-depth insights into the elemental composition, chemical state, and bonding configuration of nanoparticles incorporated onto RO membranes. These techniques offer valuable information for understanding the surface modifications, compatibility, and reactivity of the modified membranes.

XPS provides valuable information about the surface composition of modified RO membranes. By measuring the kinetic energy of photoelectrons emitted from the sample surface, XPS allows for the identification of elements present on the surface, their chemical states, and their binding energies.^150^ This information aids in determining the type and concentration of nanoparticles incorporated onto the membrane surface. Additionally, XPS can reveal changes in oxidation states, chemical functionalities, and the extent of nanoparticle dispersion. These insights are crucial for evaluating the distribution and stability of nanoparticle modifications, which impact the performance and longevity of the modified membranes. Moreover, XPS can detect subtle changes in the chemical environment surrounding the nanoparticles, providing valuable information about potential interactions with the membrane materials and the formation of chemical bonds. For instance, the XPS analysis of GO-modified PA demonstrated the formation of bonds between the TMC moieties of AP and GO NPs;^60^ however there was no clear evidence of covalent bonding between the MPD moieties of AP and GO NPs; nevertheless, it was assumed that H-bonding might be involved.^60^

FTIR spectroscopy provides information about the functional groups and molecular vibrations present in modified RO membranes. By measuring the absorption of infrared light, FTIR can identify the types of chemical bonds within the modified layer, including both surface-bound nanoparticles and the membrane matrix. Through analysis of FTIR spectra, changes in peak positions, intensities, or shifts can be correlated to chemical interactions occurring between nanoparticles and the membrane surface. These changes in chemical bonds and functional groups offer insights into modifications in surface properties, hydrophilicity, and compatibility with the aqueous environment. Furthermore, FTIR enables the identification of specific functional groups present in the modified membranes, facilitating the understanding of potential changes in surface chemistry and enabling the optimization of membrane performance. For instance, The FTIR analysis of GO-modified PA indicated that covalent bonds are indeed formed between the acyl chloride groups of TMC moieties and the functional groups of GO.^60^ This signifies a strong chemical interaction between these two components.^60^ Likewise, the presence of silica in the composite was evident from a pronounced peak in the infrared spectrum at approximately 1040–1060 cm^−1^, whose intensity increased with increasing the silica content of samples, indicating a clear correlation between the silica content and the strength of the interaction observed in the FTIR spectrum.^151^ Further, in GO-modified membranes, the existence of GO leads to peak broadening at 1585 cm^−1^ owing to combined contributions from graphene (G) band and vibrations of the polysulfone phenyl ring.^68^

The combined use of XPS and FTIR allows for a comprehensive analysis of the chemical composition and reactivity of modifications applied to RO membranes. These techniques provide detailed information about the incorporation, distribution, and stability of nanoparticles, as well as the potential modifications occurring at the membrane surface. Understanding these surface modifications and interfacial interactions is crucial for tailoring the functional properties of RO membranes, such as improved fouling resistance, enhanced flux rates, and selective ion rejection.

### 3.3 Contact angle measurement

Contact angle measurement assesses the membrane's surface energy, the wetting behavior and surface hydrophilicity or hydrophobicity of modified membranes. By placing a water droplet on the membrane surface, contact angle analysis determines the angle between the liquid–vapor interface and the solid surface. Modifications that enhance the hydrophilicity of RO membranes can improve water flux rates, reduce fouling propensity, and enhance overall membrane performance. For instance, the inclusion of GO NPs enhanced the membrane hydrophilicity, as demonstrated by a reduction in the contact angle.^60^ Namely, when 15 PA layers with 3 GO layers on top were assembled, the GO-modified PA sample (GO3/PA15) exhibited the highest GO NPs coverage on the PA surfaces and displayed the highest reduction of contact angle from 60 ± 2 to 20 ± 5 with an accompanying marginal increase in hydrophilicity.^60^ Similarly, the immobilization of Ag-NPs on the membrane led to a noticeable decrease in the contact angle, reducing it from 56.7 ± 2.2° to 32.2 ± 2.4°.^138^ In another investigation, the TFC membrane devoid of zeolite nanoparticles exhibited a relatively elevated contact angle of 68.4°.^108^ However, upon the introduction of zeolite, the contact angle of the modified membranes was reduced from 63.4° to 46.2° as the zeolite loading increased from 0.02 wt% to 0.2 wt%.^108^

### 3.4 Zeta potential analysis

The characterization of modified RO membranes using zeta potential analysis is a valuable technique in assessing the surface charge and its impact on membrane performance. Zeta potential analysis also allows the assessment of surface charge changes due to membrane modifications, such as the incorporation of nanoparticles, functional groups, or surface coatings, all of which can alter the surface charge, and result in improved anti-fouling properties or enhanced selectivity. A higher magnitude of zeta potential indicates a greater electrostatic repulsion between the membrane surface and charged solutes, reducing fouling potential and promoting higher rejection of dissolved species. Furthermore, monitoring zeta potential changes over time can help assess the stability and durability of modified RO membranes. Variations in zeta potential can indicate changes in membrane properties, such as fouling accumulation or chemical degradation, providing valuable information for membrane maintenance and cleaning protocols. For instance, incorporation of 0.1 weight percent of multi-walled carbon nanotubes (MWCNTs) reduced the modified membrane's zeta potential and surface charge.^73^

### 3.5 Thermal stability

Thermogravimetric Analysis (TGA) and Differential Scanning Calorimetry (DSC) are two key techniques used in the characterization of nanoparticles-modified TFC membranes for reverse osmosis (RO) applications. These techniques provide valuable information on the thermal stability, decomposition behavior, and phase transitions of nanocomposite films as a function of temperature. During a typical TGA experiment, the sample is subjected to a controlled temperature ramp, and as the temperature increases, the weight loss due to volatilization, degradation, or combustion of the constituents is recorded. TGA enables the determination of the temperature at which weight loss begins, which indicates the onset of decomposition or degradation. Moreover, DSC is a technique used to analyze the thermal properties, phase transitions, and heat flow characteristics of materials as a function of temperature.^152^ It measures the difference in heat flow to or from a sample and a reference during controlled heating or cooling. This technique gives valuable data about melting temperature (*T*_m_) glass transition temperature (*T*_g_), and crystallization temperature (*T*_c_) of various polymer types.^152,153^ For instance, using TGA to examine the thermal stability of TFC and TFN membranes revealed that the distribution of TiO_2_ NPs within the TFN membrane, produced a slightly higher onset temperature for intense degradation compared to the unmodified TFC membrane.^127^ Namely, the onset degradation temperature increased from 530 to 550 °C upon the incorporation of TiO_2_ NPs.^127^ Similarly, the TGA curves provided evidence supporting the correlation between the addition of SiO_2_ and a reduction in thermal decomposition. The results indicated that as the SiO_2_ content increased, the thermal decomposition of modified membranes decreased.^151^

Overall, thermal stability data allows the determination of the degradation temperature, which is the temperature at which a significant weight loss occurs. This information helps in assessing the temperature limits that the nanocomposite film can tolerate during RO operations, ensuring its stability and preventing any detrimental effects on the film's properties.

## 4. Challenges in TFC membrane

Most research papers of TFN membranes focus on membrane modification with a given nanomaterial to prepare a novel membrane and study its improved performance characteristics. However, there is a need for a systematic monovariate exploration of incorporating one nanomaterial with various membrane preparation methods and in various locations to produce TFNa, TFNs and TFNi containing the same controlled size, porosity, and hydrophilicity NPs. An additional challenge is maximizing TFN membranes' performances while incorporating different nanomaterials with different features in various membrane positions *via* regulating the IP process.^39–41^ The primary challenge in incorporating nanomaterials in polyamide membrane fabrication is achieving a uniform dispersion of the nanofillers within the polymer matrix.^154,155^ Poor dispersion can lead to accumulation, uneven properties, and reduced membrane performance.^33^ Factors such as the surface chemistry of nanomaterials, shear forces during processing, and compatibility with the polymer matrix all influence dispersion quality. For instance, the dispersion of hydrophilic nanoparticles in non-polar chemical solvents is unstable.^156^ It is challenging for large hydrophilic nanoparticles in water to phase-transfer to a nonpolar organic solvent.^156^ Researchers are currently focusing on surface modification approaches for nanomaterials, including the employment of amine-functionalized or carboxylated carbon nanotubes (CNTs) and halloysite nanotubes (HNTs).^156–159^ Modifying the surface properties of nanoparticles can help minimize agglomeration in nonpolar solvents or polymer matrices.^33^ In addition to modifying the surface of nanofillers, novel nanomaterials such as MOFs and COFs can be created with specific pore structure and surface charge to promote even dispersion of nanofillers within the polymer matrix. Moreover, the intrinsic orientation of 1D and 2D nanofillers during the IP processes can be another challenge, *e.g.*, most nanotubes because of gravitational forces prefer horizontal alignment, creating longer paths and higher resistance for horizontal nanochannels water flow. Otherwise, nano-bowls that are randomly dispersed create extra resistance for water flow.^40,159,160^ Additionally, horizontal nanochannels of nanosheets can be disrupted with long pressurization, and lead to TFN membrane performance deterioration. It is common knowledge that the longer the path, the greater the hydraulic resistance. 2D nanomaterials like COF have internal channels that allow water molecules to flow through with reduced distance, making them advantageous compared to traditional materials.^40^ Hence, the concept of the preferential pathway for water molecules requires further verification since it is only possible if the nanochannels are aligned toward the water flux direction and not blocked by the polymer matrix. So far, most studies on liquid separation reported the membrane performance results with only randomly arranged nanotubes. Furthermore, the trade-off between water permeability, salt rejection rate, fouling and Cl_2_-resistance should be carefully considered. The nanofillers toxicity and appreciable leaching rates from TNF membranes should also be avoided. Moreover, green synthesis of nanofillers and TFN membranes are preferred. Another significant challenge in incorporating nanomaterials in polyamide membrane fabrication is ensuring the stability and durability of the composite membrane under harsh operating conditions. Nanomaterials, such as carbon nanotubes, graphene oxide, or metal nanoparticles, can be susceptible to degradation, oxidation, or leaching, which can compromise the long-term performance of the membrane. Finally, the scalability and cost-effectiveness of incorporating nanomaterials in polyamide membrane fabrication are critical factors that need to be considered to meet industrial production requirements. Advanced manufacturing processes, such as electrospinning, 3D printing, and roll-to-roll coating, can be employed to scale up the production of nanocomposite membranes while maintaining cost-effectiveness.^40,159,160^

## 5. Economic analysis

**Fig. 15:**
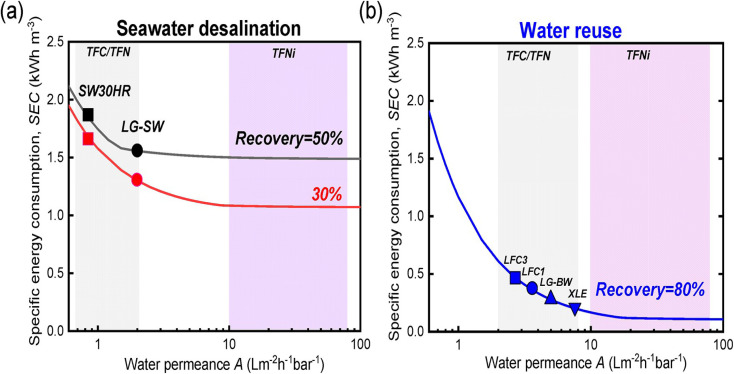
Relationship between specific energy consumption (SEC, kW h m^−3^) and product flow in the context of (a) seawater desalination and (b) water reuse. Reprinted with the permission of ref. 29, copyright 2024, American Chemical Society.

The economic aspects of modified RO membranes incorporating nanomaterials (graphene, CNTs, MOF, zeolites, TiO_2_, and SiO_2_, Ag NPs, *etc.*) for water treatment applications are crucial considerations in determining their feasibility and commercial viability. Nanomaterial-modified RO membranes offer improved performance characteristics, such as increased permeability, salt rejection, reduced cleaning requirements, antimicrobial properties, and enhanced mechanical, chemical, and physical stability. These advancements translate into several economic benefits, including higher water production rates, reduced energy consumption, improved water quality, and extended membrane lifespan. The increased productivity and efficiency of nanomaterial-modified membranes can result in cost savings over time, making them economically viable alternatives to traditional membranes. A research^161^ highlighted the important role of membrane modification in lowering specific energy consumption (SEC) during desalination processes across different water sources (Fig. 15a and b). The study demonstrated considerable energy savings of up to 80% in scenarios involving feed waters with low osmotic pressure, such as water reuse. However, the cost of nanomaterials is a significant consideration when assessing their economic feasibility. Additionally, nanomaterials may have limited availability and higher market costs due to their specialized nature and manufacturing constraints. These higher initial costs can pose a barrier to the widespread adoption of nanomaterial-modified membranes, particularly in large-scale applications.

For instance, metal oxides, silica, and zeolites are generally more economically viable due to their wide availability and competitive pricing. The use of metal oxides in RO membrane modification provides several economic advantages. They are readily available in bulk quantities, ensuring a steady supply and competitive pricing. Furthermore, the relatively low material costs associated with metal oxides make them economically viable for large-scale applications. The cost-effectiveness of these materials is particularly advantageous for water treatment facilities and industrial processes where long-term cost savings are essential. Similarly, zeolite nanoparticles are relatively cost-effective and readily available in the market, making them a cost-effective option for membrane modification.

On the other hand, carbon-based nanomaterials, namely graphene, single-walled carbon nanotubes (SWCNTs), and multi-walled carbon nanotubes (MWCNTs), hold significant potential for enhancing RO membrane performance. Their unique properties, including high permeability, hydrophilicity, and salt rejection rates, make them ideal candidates for improving desalination efficiency. However, the economic feasibility of carbon nanomaterials remains a challenge. The high cost of production and limited availability hinder their widespread adoption. In addition, the complex manufacturing processes required for these nanomaterials make large-scale production and local availability difficult. Further research and development are necessary to overcome these economic barriers and make carbon nanomaterials more cost-effective. Further, the use of silver coatings in RO membrane modification offers antimicrobial properties, effectively preventing biofouling. This reduces the need for frequent membrane cleaning and maintenance, leading to long-term cost savings. While silver is relatively more expensive compared to other materials, the potential economic benefits resulting from reduced cleaning requirements should be considered. Moreover, the integration of these nanomaterials into the RO membranes involves additional processing steps, which might increase manufacturing costs. Techniques such as physical blending, electrospinning, and chemical deposition methods require specific instrumentation, energy consumption, and skilled labor. Hence, an economic analysis could provide insights into the long-term monetary benefits of employing these modified membranes in terms of reduced operational expenses.

## 6. Conclusion and prospective

In conclusion, the incorporation of nanomaterials into RO membranes holds great promise for improving membrane performance and addressing challenges in water purification. Utilizing inorganic and porous organic material, TFN membranes can be fabricated through interfacial polymerization by dispersing NPs in the organic phase or the aqueous solution. The inclusion of nanomaterials in the PA layer not only creates nanochannels for enhanced water transport and consistent solute rejection but also changes membrane features such as hydrophilicity, surface roughness, cross-linking degree, and surface charge, which significantly impact membrane performance. Extensive research studies have demonstrated the positive impacts of these nanomaterials on various membrane features, including rejection rates, water flux, selectivity, chlorine resistance, and fouling resistance. The optimal dose of nanomaterials and suitable fabrication methods play vital roles in achieving the overall desired performance. Incorporating materials like SiO_2_ further enhances the mechanical strength and durability of the membrane, thereby extending its lifespan. Likewise, membranes containing CNTs, GO, SiO_2_ and TiO_2_ NPs exhibit versatility in RO applications due to their exceptional thermal, electrical, fouling resistance and mechanical stability, as well as their antibacterial and photocatalytic features. Additionally, it is worth noting that MOFs are particularly attractive porous nanomaterials that warrant intensive research attention in the fabrication of TFN membranes. However, there are still areas of concern and knowledge gaps in the existing literature that will require further investigation in the future:

1. Tailored material design: future research can focus on developing and tailoring carbon nanomaterials, and other NPs with specific properties for membrane modification. This could involve exploring new synthesis methods, optimizing material composition, and engineering advanced structures at the nanoscale. By fine-tuning material properties, such as surface chemistry, pore size, porosity, and hydrophilicity, researchers can enhance membrane selectivity, permeability, and fouling resistance.

2. Mechanistic insights: gaining a deeper understanding of the mechanisms underlying the interaction between modified membranes and various pollutants is crucial for optimizing membrane performance.

3. Scaling up and commercial viability: to enable the practical implementation of NPs-modified membranes, researchers need to address scalability and cost-effectiveness. Further research should focus on developing scalable and cost-efficient synthesis methods for incorporating nanomaterials into large-area membranes. The durability and stability of modified membranes under realistic operating conditions must also be thoroughly evaluated to ensure long-term performance. Collaborations between academia and industry can facilitate the translation of research findings into commercially viable membrane products.

4. Environmental sustainability: as the field progresses, it is crucial to consider the broader environmental implications of modified membranes. Research should explore the life cycle analysis of these membranes, including the environmental impact of raw material extraction, synthesis processes, and end-of-life disposal. Additionally, efforts should be made to develop recycling and regeneration methods for used membranes to minimize waste generation.

Overall, the use of nanomaterials to modify reverse osmosis membranes holds promise for advancing membrane technology and addressing challenges in water treatment. Continued research and development in this field will contribute to the improvement of membrane science and play a critical role in providing sustainable and efficient solutions for water purification and desalination processes on a global scale.

## Conflicts of interest

Authors declare no conflict of interest.

## Supplementary Material
